# Similarities and Differences in Decision-Making Impairments between Autism Spectrum Disorder and Schizophrenia

**DOI:** 10.3389/fnbeh.2015.00259

**Published:** 2015-09-23

**Authors:** Long Zhang, Jiulai Tang, Yi Dong, Yifu Ji, Rui Tao, Zhitu Liang, Jingsong Chen, Yun Wu, Kai Wang

**Affiliations:** ^1^Department of Neurology, The First Affiliated Hospital of Anhui Medical University, Hefei, China; ^2^Laboratory of Neuropsychology, Anhui Medical University, Hefei, China; ^3^Department of Children Rehabilitation, The First Affiliated Hospital of Anhui Medical University, Hefei, China; ^4^Mental Health Center of Anhui Province, Hefei, China; ^5^Hefei Chunya Mutual Association, Hefei, China; ^6^Department of Rehabilitation, Hefei Jingu Hospital, Hefei, China; ^7^Department of Psychology, Peking University, Beijing, China

**Keywords:** autism spectrum disorder, decision-making, decision-making under ambiguity, decision-making under risk, Game of Dice Task, Iowa Gambling Task, schizophrenia

## Abstract

Although individuals with autism spectrum disorders (ASD) and schizophrenia (SCH) share overlapping characteristics and may perform similarly on many cognitive tasks, cognitive dysfunctions common to both disorders do not necessarily share the same underlying mechanisms. Decision-making is currently a major research interest for both ASD and SCH. The aim of the present study was to make direct comparisons of decision-making and disorder-specific underlying neuropsychological mechanisms between the two disorders. Thirty-seven participants with ASD, 46 patients with SCH, and 80 healthy controls (HC) were assessed with the Iowa Gambling Task (IGT), which measures decision-making under ambiguity, and the Game of Dice Task (GDT), which measures decision-making under risk. The results revealed that both the ASD and SCH groups had deficits for both the IGT and the GDT compared with the HC. More importantly, in the IGT, participants with ASD displayed a preference for deck A, indicating that they had more sensitivity to the magnitude of loss than to the frequency of loss, whereas patients with SCH displayed a preference for deck B, indicating that they showed more sensitivity to the frequency of loss than to the magnitude of loss. In the GDT, the impaired performance might be due to the deficits in executive functions in patients with SCH, whereas the impaired performance might be due to the deficits in feedback processing in participants with ASD. These findings demonstrate that there are similar impairments in decision-making tasks between ASD and SCH; however, these two disorders may have different impairment mechanisms.

## Introduction

Although autism spectrum disorders (ASD) and schizophrenia (SCH) are classified as mutually exclusive diagnoses in the most recent revision of the *Diagnostic and Statistical Manual* (DSM-5) (APA, 2013), genetic studies have revealed that there is an overlap in genetic risk between the two disorders (Ionita-Laza et al., 2014; McCarthy et al., 2014). Similarly, many studies have indicated that these two disorders share overlapping characteristics (Mealey et al., 2014). Impairment in social functions is one of the primary features of the clinical presentations of both ASD and SCH (Couture et al., 2010). A range of impairments in social cognition have been reported in subjects with both ASD and SCH, including impairments in face processing (Sasson et al., 2007; Sachse et al., 2014), theory of mind (Chung et al., 2014), decision-making (Brown et al., 2015; Mussey et al., 2015), and empathy (Lugnegård et al., 2013).

Decision-making, one of the most frequently investigated social functions, refers to the process of striking a balance between a set of alternative options with different likelihoods of reward and punishment (Cheng et al., 2012). Aberrant and maladaptive decision-making has been described as a key concept in understanding several behavioral disturbances in different types of psychiatric and neurological disorders, including ASD and SCH (Brand et al., 2005; Fond et al., 2013; Mussey et al., 2015). As the neural and cognitive mechanisms of decision-making are better understood, there is a greater potential of revolutionizing the nosology, diagnosis, and treatment of these disorders (Lee, 2013).

From a neuroscientific perspective, there are at least two types of decision-making that differ primarily in their degrees of uncertainty and the amount of useful information provided about possible consequences and their probabilities (Brand et al., 2006). In some situations, outcomes and probabilities are implicit, and the decision makers must initially find useful information and determine the options’ qualities by means of processing feedback of previous choices. This type of decision-making is often termed decision-making under ambiguity and is usually measured with the Iowa Gambling Task (IGT; Bechara et al., 2000). In this task, participants are presented with four decks and series of cards from which they must make choices. They are unaware of the quantity of cards they need to choose or which decks are disadvantageous (i.e., coupling large gains with even larger losses, which leads to a negative overall balance in the long term) or advantageous (i.e., coupling small gains with even smaller losses, which leads to a positive overall balance in the long term).

In general, decision-making seems to be influenced differently by various parameters, such as the magnitudes/frequencies of gains/losses, response costs, delays, and probabilities of gains/losses (Drechsler et al., 2010). An important factor that might contribute to performance on the IGT is individual deck level preferences. The role of individual deck level preferences in assessing IGT performance was overlooked by the majority of the previous studies (Buelow and Suhr, 2013, 2014). Although decks A and B lead to long-term negative outcomes, deck A includes high-frequency and low-magnitude losses while deck B includes low-frequency and high-magnitude losses. Greater deck A or greater deck B selections depend on whether the participants display more sensitivity to the magnitudes or frequencies of losses (Bechara, 2008).

In contrast to decision-making under ambiguity, explicit information about the potential consequences of various choices and their probabilities are provided in some decision situations. This type of decision-making is referred to as decision-making under risk, and it is frequently measured with the Game of Dice Task (GDT; Brand et al., 2005). The GDT requires subjects to choose between alternative categories that are explicitly related to a specific amount of gain/loss. Winning probabilities are obvious and stable from the beginning of the task. Two out of the four alternative categories related to high potential gains/losses but low probabilities of winning are high-risk decisions; the other two alternative categories related to lower potential gains/losses but higher probabilities of winning are low-risk decisions. Thus, subjects are able to estimate the risk related to each alternative category and apply strategies to maximize their profits.

Previous studies have shown that performance on the GDT significantly correlates with executive functions, such as set-shifting, categorization, and cognitive flexibility as measured by the Wisconsin Card Sorting Task (WCST). The relationship between them was confirmed not only in healthy controls (HC) (Gathmann et al., 2014; Schiebener et al., 2014) but also in participants with various neuropsychiatric disorders (Brand et al., 2005; Labudda et al., 2009). In addition, several studies have related impaired decision-making in the GDT to poor capacities to advantageously utilize feedback processing (Brand, 2008; Yao et al., 2014). For instance, participants who use negative feedback, in terms of losses, to modify their strategies appear to choose the low-risk options more frequently than those who ignore receiving losses, and they are more likely to obtain a better performance on the GDT (Labudda et al., 2009).

There were a series of studies in which the IGT and GDT were used to examine decision-making performance in participants with ASD and patients with SCH. Four previous studies that used the IGT investigated decision-making under ambiguity in participants with ASD. Two studies found that both the patient group and the control participants learned to make more advantageous decisions during the first half of the IGT; however, toward the second half of the task, the participants with ASD did not continue to increase their choices from advantageous options at the same rate as the control subjects (Johnson et al., 2006; Yechiam et al., 2010). Both studies suggested that the participants with ASD might have made more frequent shifts between decks during the IGT and had difficulty learning which alternatives were advantageous and which were disadvantageous. Another study by Mussey et al. (2015) indicated that the participants with ASD displayed impaired performance on the IGT and slower learning with regard to which decks were advantageous compared to the HC. In contrast, South et al. (2014) observed the opposite pattern. They found that subjects with ASD performed better on the IGT than a group of control participants. Previous studies have investigated the performance of patients with SCH on the IGT; these yielded inconsistent results. On the one hand, several studies have shown that the performance of patients with SCH does not differ from that of controls (Cavallaro et al., 2003; Rodriguez-Sanchez et al., 2005). On the other hand, most studies have found that SCH patients choose more disadvantageous options than healthy subjects (Lee et al., 2007; Sevy et al., 2007; Yip et al., 2009; Raffard et al., 2011; Cella et al., 2012; Brown et al., 2015).

To the best of our knowledge, no study so far has investigated decision-making under risk using the GDT in participants with ASD. Only two studies have examined decision-making in SCH patients using the GDT. One revealed that there was no significant difference in GDT performance between SCH patients and HC (Lee et al., 2007). Another study showed that patients with SCH demonstrated inferior performance on the GDT as compared to HC. It also showed that they differed from HC in that they made a higher number of high-risk choices (Fond et al., 2013).

A series of studies that independently examined decision-making hinted at significant overlap between these two disorders. Both patient groups have demonstrated impaired performance on decision-making tasks compared to HC (Raffard et al., 2011; Brown et al., 2015; Mussey et al., 2015). However, no study to date has attempted to compare the decision-making performance of individuals with ASD to the performance of patients with SCH. Furthermore, it is important to note that while decision-making dysfunctions common to both disorders may be phenotypically similar, they do not necessarily share the same underlying mechanism (Mealey et al., 2014). For instance, to identify disorder-specific underpinnings of emotion processing in ASD and paranoid SCH, Sachse et al. (2014) compared facial emotion recognition abilities on different levels of emotion and stimuli complexity. These researchers’ findings revealed distinct underlying neurocognitive abilities in each patient group compared with HC, with poorer face identity recognition in individuals with ASD and reduced visual perception in patients with SCH.

Accordingly, we investigated decision-making in carefully diagnosed and well-characterized samples of participants with ASD and SCH using the IGT, the GDT, and a neuropsychological test battery and compared them to HC. Consistent with the studies discussed, we hypothesized that both participants with ASD and SCH would display poorer performance on the IGT and the GDT compared to a matched control group. Additionally, we further investigated whether there were disorder-specific underlying cognitive mechanisms for impaired performance on the IGT and the GDT in these two groups.

## Materials and Methods

### Participants

The study sample included 37 participants with ASD, 46 patients with SCH, and 80 HC. The diagnostic assessments of the patients were initially performed by two experienced psychiatrists and were confirmed using the Structural Clinical Interview for DSM-IV-TR (APA, 2000). All of the participants gave written informed consent. The study was executed in accordance with the Declaration of Helsinki, and it was approved by the ethical committee at Anhui Medical University.

The participants with ASD were recruited from the outpatients of the Department of Rehabilitation at the First Affiliated Hospital of Anhui Medical University. The participants with ASD had been diagnosed with Asperger’s disorder or high-functioning autism according to the DSM-IV-TR (APA, 2000). According to the DSM-5 (APA, 2013), individuals with a well-established DSM-IV-TR diagnosis of autistic disorder, Asperger’s disorder, or pervasive developmental disorder – not otherwise specified – should be given the diagnosis of ASD. Thus, the participants were diagnosed as having ASD. The participants were excluded if they: (1) met any other DSM-IV-TR axis I diagnosis, (2) had been treated with any psychiatric medication or (3) had an IQ score lower than 75. Standardized diagnostic scales such as the Autism Diagnostic Observation Schedule (Lord et al., 2000) and the Autism Diagnostic Interview – Revised (Lord et al., 1994) have not been adapted for use in mainland China. To confirm the diagnosis of ASD, all of the participants completed the Chinese version of the Autism-Spectrum Quotient (AQ; Baron-Cohen et al., 2001) to corroborate their clinical presentation. Participants with ASD scored above the cutoff point. In addition, the IQ scores of all of the participants were tested using the standardized Raven test (Wang and Qian, 1997). The ASD group totaled 37 subjects, which included 6 women and 31 men (mean age: 18.9 ± 3.64 years; years of education: 8.7 ± 2.8; IQ: 103.2 ± 14.6).

The SCH patients were recruited from the Mental Health Center of Anhui Province and were hospitalized at the time of the assessment. SCH patients were included if they: (1) met the DSM-IV-TR diagnostic criteria for first-episode SCH, (2) did not meet any other DSM-IV-TR axis I diagnoses, and (3) had never been treated with any antipsychotic medications. The SCH group totaled 46, consisting of 9 women and 37 men (mean age: 19.9 ± 3.76 years; years of education: 10.5 ± 1.8; IQ: 106.5 ± 16.0). Symptom severity was assessed with the Positive and Negative Syndrome Scale (PANSS) for SCH (PANSS total: 94.8 ± 9.4; PANSS positive: 25.6 ± 5.9; PANSS negative: 20.7 ± 4.4; PANSS general psychopathology: 48.5 ± 4.1). In addition, the SCH patients’ AQ scores were significantly below the cutoff score.

Participants without a history of psychiatric illness or a known family history of ASD or SCH were recruited as HC through advertisements and leaflets or by word of mouth among college students and the local community. The exclusion criteria were current or past diagnoses of psychiatric disorders, neurological illnesses, drug or alcohol abuse, gambling addictions, or serious medical illnesses. The HC group totaled 80 participants: 13 women and 67 men (mean age: 19.2 ± 2.96 years; years of education: 10.6 ± 1.4; IQ: 108.1 ± 14.3). The AQ scores of the HC were significantly below the cutoff score. There were no significant differences in age [*F*(2,160) = 1.00, *p* = 0.371], sex (χ^2^ = 0.26, df = 2, *p* = 0.880), or IQ [*F*(2,160) = 1.06, *p* = 0.348] among the three groups.

### Neuropsychological background tests

#### Wisconsin Card Sorting Task

The WCST (Heaton et al., 1993), which measures executive function, organization, and set-shifting, consists of four different types of stimulus cards. Participants are given a set of target cards and requested to detect sorting principles and to match each target card with one of the four stimulus cards. However, the sorting pattern changes after 10 sequential correct responses and participants must switch to a new sorting pattern based on the feedback. The total sum of error responses, the total sum of perseverative responses, the total sum of perseverative errors, and the total sum of categories completed are calculated for analyses. Participants that score low in the first three variables or score high in the last variable have a better capacity in cognitive flexibility, categorization, and set-shifting.

#### Trail Making Test

For Trail Making Test (TMT) A, subjects were asked to connect 25 encircled numbers as accurately and quickly as possible in ascending order. For TMT B, subjects were asked to connect numbers and letters alternately (e.g., 1, A, 2, B, 3, C). TMT A measures mental tracking and motor speed, and TMT B captures selective attention and cognitive flexibility (Reitan, 1992). The amount of time required to complete each test was calculated for analyses. A shorter time needed for the TMT indicates better performance. We also calculated the time difference (TMT B − TMT A) by subtracting the time required to complete TMT A from the time required to complete TMT B. The time difference is calculated to remove the speed component from the test evaluation, with high difference scores indicating that participants have problems with multiple tracking (Lezak et al., 2004).

#### Digit Span Test

The Digit Span Test (DST) (Wechsler, 1987) is a common measure of verbal short-term memory and verbal working memory. In the DST, participants are asked to repeat a sequence of different numbers either forward or backward. The memory span corresponds to the largest serial of numbers repeated in the right order. Two scores were collected: the digit span forward and backward scores. The more participants repeat, the better verbal short-term memory and verbal working memory participants have.

### Decision-making under ambiguity

The computerized version of the IGT was used to measure decision-making under ambiguity (Bechara et al., 2000). In this task, subjects are instructed to choose one card from four decks of cards (A, B, C, and D). After each card selection, they win or lose a specified amount of money. On the IGT, decks A and B yield an average gain of €100 per selection, and decks C and D yield an average gain of €50 per selection. Ten selections from decks A or B lead to a net loss of €250, whereas 10 selections from decks C or D lead to a net gain of €250. In short, A and B are disadvantageous decks, they include high immediate gains, but even higher losses, resulting in a negative outcome over the long run; decks C and D are advantageous, they produce small immediate gains, but even smaller losses, resulting in a positive outcome in the long term. Moreover, there are also other inequalities between the four decks. For instance, although decks A and B lead to long-term negative outcomes, selections from deck A are punished on 50% of trials but deck B selections are punished on 10% of trials. The immediate losses on deck A are also smaller than those in deck B. Similar differences are observed between decks C (50% losses) and D (10% losses), and the immediate losses in deck C are also smaller than those in deck D (Bechara, 2008).

Subjects are told to increase their starting capital of €2000 by winning as much money as possible. The IGT involves 100 card selections, but subjects are unaware that they have to select 100 cards. To analyze task performance, we calculated the total net score by subtracting the number of disadvantageous choices from the number of advantageous choices. A positive net score indicates profitable decision-making performance. The 100 trials were divided into five equal blocks, and the net score of each block of 20 cards was calculated to investigate whether decision-making changed during the task. Furthermore, the number of cards selected in individual decks A, B, C, and D was calculated to examine the individual deck level preference.

### Decision-making under risk

To assess decision-making under risk, we used the computerized GDT (Brand et al., 2005). The goal of the GDT is to increase the starting capital of €1000 within 18 trials. Participants are asked to bet before each trial which number will be thrown next. This can be done either by betting on a single number or a combination of two, three, or four numbers. They win some money if the chosen number or one of the chosen numbers is thrown, otherwise they lose the same amount of money. Each alternative category is associated with defined gain/loss and different winning probabilities: €1000 gain/loss with a winning probability of 1:6 for a single number (single choice); €500 gain/loss with a winning probability of 2:6 for combination of two numbers (double choice); €200 gain/loss with a winning probability of 3:6 for combination of three numbers (triple choice); €100 gain/loss with a winning probability of 4:6 for combination of four numbers (quadruple choice). The two former alternative categories, which have lower winning probabilities, are grouped into high-risk decisions; the two latter alternative categories, which have higher winning probabilities, are grouped into low-risk decisions. For analysis, we calculated a net score (the number of low-risk choices − the number of high-risk choices) to analyze task performance. A positive net score indicates profitable decision-making performance. We also calculated how often the four different alternative categories (single number, two, three, or four numbers) were chosen.

### Statistical analyses

SPSS 16.0 was used to perform all of the statistical analyses. All of the variables were tested for normal distribution with the Kolmogorov–Smirnov test separately for the three groups. There were no significant deviations from the normal distribution for the IGT net score, the GDT net score, or the neuropsychological variables. Thus, parametric methods were used for these variables. A one-way analysis of variance (ANOVA) with group as the between-subject factor was performed to examine the IGT net score and GDT net score. A one-way ANOVA with block as the between-subject factor was performed to examine the influence of the decision process on the IGT net score. A repeated-measures ANOVA with block as the within-subject factor and group as the between-subject factor was performed to compare the net score of the five blocks across the groups. A one-way ANOVA with group as the between-subject factor, a one-way ANOVA with deck as the between-subject factor, and a repeated-measures ANOVA with group as the between-subject factor and deck as the within-subject factor were performed to examine individual deck level preferences between groups. A repeated-measures ANOVA with deck as the between-subject factor and block as the within-subject factor was performed to examine how the individual deck level preference changes over the IGT. An ANOVA with repeated measures with alternative category as the within-subject factor and group as the between-subject factor was conducted to compare the difference of selection in the four alternative categories across the groups. Additionally, we used a Pearson correlation analysis to examine the correlation between the neuropsychological test scores and the decision-making scores. The threshold of statistical significance was set at *p* < 0.05.

## Results

### Neuropsychological assessment

The three groups differed in the WCST (see Table 1). According to the *post hoc* Bonferroni-corrected comparisons, the SCH group performed significantly worse than the ASD and HC groups in the total errors, perseverative errors, and categories completed (all *p*s < 0.05, all *d*s ≥ 0.45), and there were no significant differences between the ASD and HC groups (all *p*s > 0.05, all *d*s ≤ 0.20). Additionally, the three groups differed in the TMT (see Table 1). According to the *post hoc* Bonferroni-corrected comparisons, the ASD and HC groups performed significantly better on the TMT B and difference score (TMT B − TMT A) (all *p*s < 0.05, all *d*s ≥ 0.40), and there were no significant differences between the ASD and HC groups (all *p*s > 0.05, all *d*s ≤ 0.13). No differences were found between the three groups for the DST (all *p*s > 0.05), indicating that performance of the ASD and SCH groups on the DST was comparative with that of the HC group.

**Table 1:** **Results of the neuropsychological tasks [mean (SD)]**.

	ASD (*n* **=** 37)	SCH (*n* **=** 46)	HC (*n* **=** 80)	*F*	*p*	df
**WCST**						
Total errors	38.70 (19.80)	46.80 (16.03)	37.91 (16.50)	4.21	0.017	2,160
Perseverative response	45.78 (25.58)	50.74 (23.62)	49.72 (25.25)	0.45	0.636	2,160
Perseverative errors	21.16 (13.81)	30.89 (12.73)	24.08 (14.78)	5.58	0.005	2,160
Categories completed	5.97 (2.36)	4.61 (2.01)	5.66 (2.52)	4.20	0.017	2,160
**TMT**						
TMT A (s)	33.39 (6.33)	34.82 (5.55)	34.90 (6.00)	0.89	0.415	2,160
TMT B (s)	66.97 (11.48)	73.15 (8.99)	69.41 (9.69)	4.17	0.017	2,160
TMT B − TMT A (s)	33.58 (11.13)	38.34 (7.98)	34.52 (9.13)	3.34	0.038	2,160
**DST**						
DST forward	9.16 (1.66)	9.72 (1.49)	9.42 (1.49)	1.37	0.257	2,160
DST backward	6.32 (1.58)	6.02 (1.13)	6.25 (1.35)	0.62	0.542	2,160

*ASD, autism spectrum disorders; SCH, schizophrenia; HC, healthy controls; WCST, Wisconsin Card Sorting Test; TMT, Trail Making Test; DST, Digit Span Test*.

### Decision-making on the IGT

#### Net Score on the IGT

**Figure 1:**
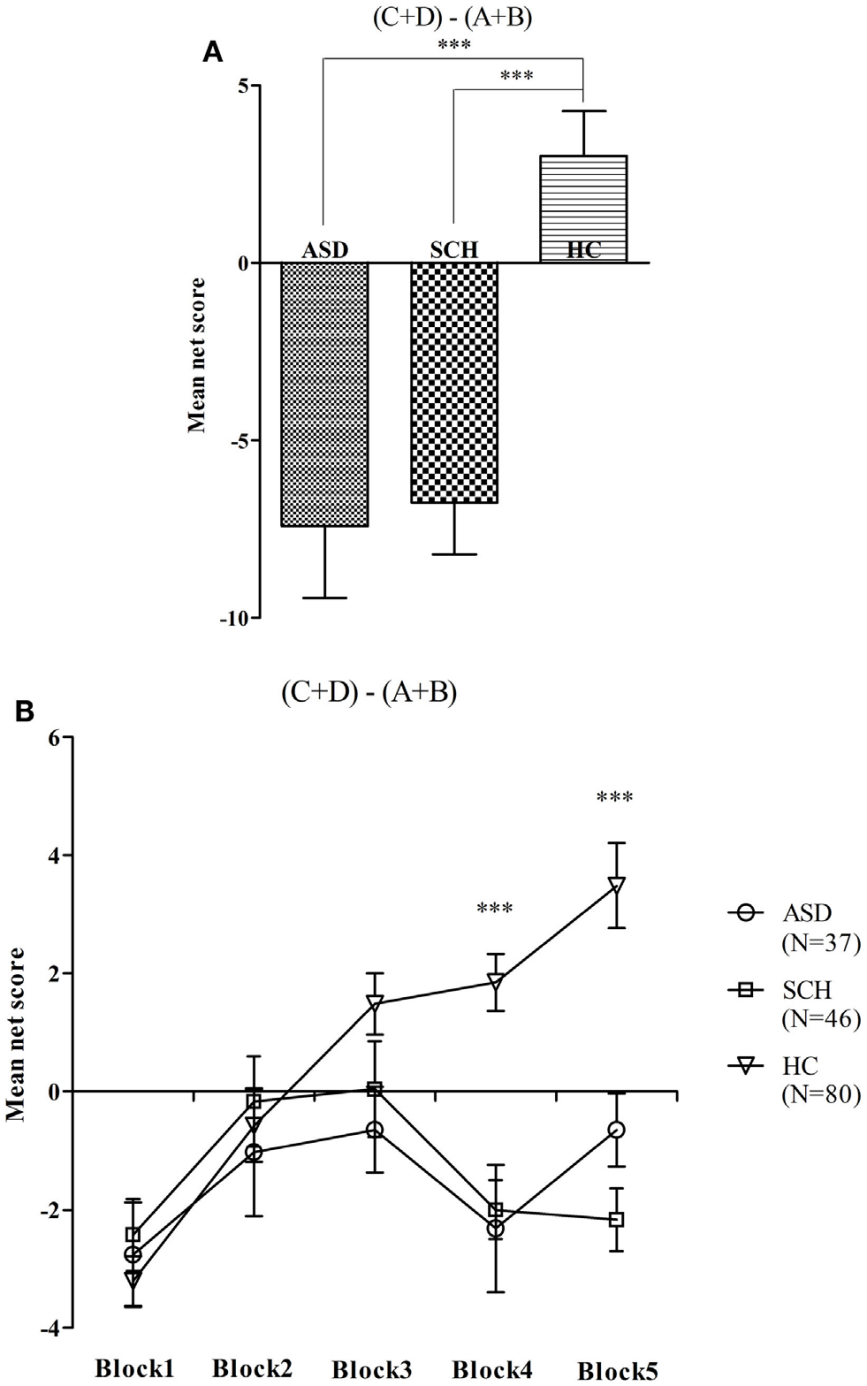
**Mean net score in the IGT (A) and mean net score for each block of 20 trials (B) for participants with ASD, SCH, and HC**. ***p < 0.001. Mean ± SEM is shown.

There were significant differences between net score on the IGT in the three groups [*F*(2,160) = 16.62, *p* < 0.001]. According to the *post hoc* Bonferroni-corrected comparisons, the HC group scored higher than the ASD and SCH groups (all *p*s < 0.001, all *d*s ≥ 0.40), and there was no significant difference between the ASD and SCH groups (*p* = 1.000, *d* = 0.03) (Figure 1A). To compare the net score of the five blocks across the groups, a repeated-measures ANOVA was performed with block as the within-subject factor and group as the between-subject factor. There were significant main effects for group [*F*(2,160) = 16.62, *p* < 0.001], and for block [*F*(4,640) = 9.34, *p* < 0.001], and for group by block interaction effects [*F*(2,160) = 16.31, *p* < 0.001]. The comparisons of performance on the five blocks indicated that the three groups scored differently in block 4 [*F*(2,160) = 14.85, *p* < 0.001]. According to the *post hoc* Bonferroni-corrected comparisons, the HC group scored higher than the ASD and SCH groups (all *p*s < 0.001, all *d*s ≥ 0.75), and there was no significant difference between the ASD and SCH groups (*p* = 1.000, *d* = 0.06). The three groups also scored differently in block 5 [*F*(2,160) = 19.24, *p* < 0.001]. According to the *post hoc* Bonferroni-corrected comparisons, the HC group scored higher than the ASD and SCH groups (all *p*s < 0.001, all *d*s ≥ 0.78), and there was no significant difference between the ASD and SCH groups (*p* = 0.566, *d* = 0.41) (Figure 1B). That is, the HC group performed significantly better on the IGT than the ASD and SCH groups.

#### Individual Deck Level Preference

A repeated-measures ANOVA with group as a between-subject factor and deck as a within-subject factor was performed. There was a significant main effect for deck [*F*(3,480) = 26.90, *p* < 0.001], no significant main effect for group [*F*(2,160) = 1.00, *p* = 0.369], and an interaction effect [*F*(3,480) = 11.63, *p* < 0.001].

**Figure 2:**
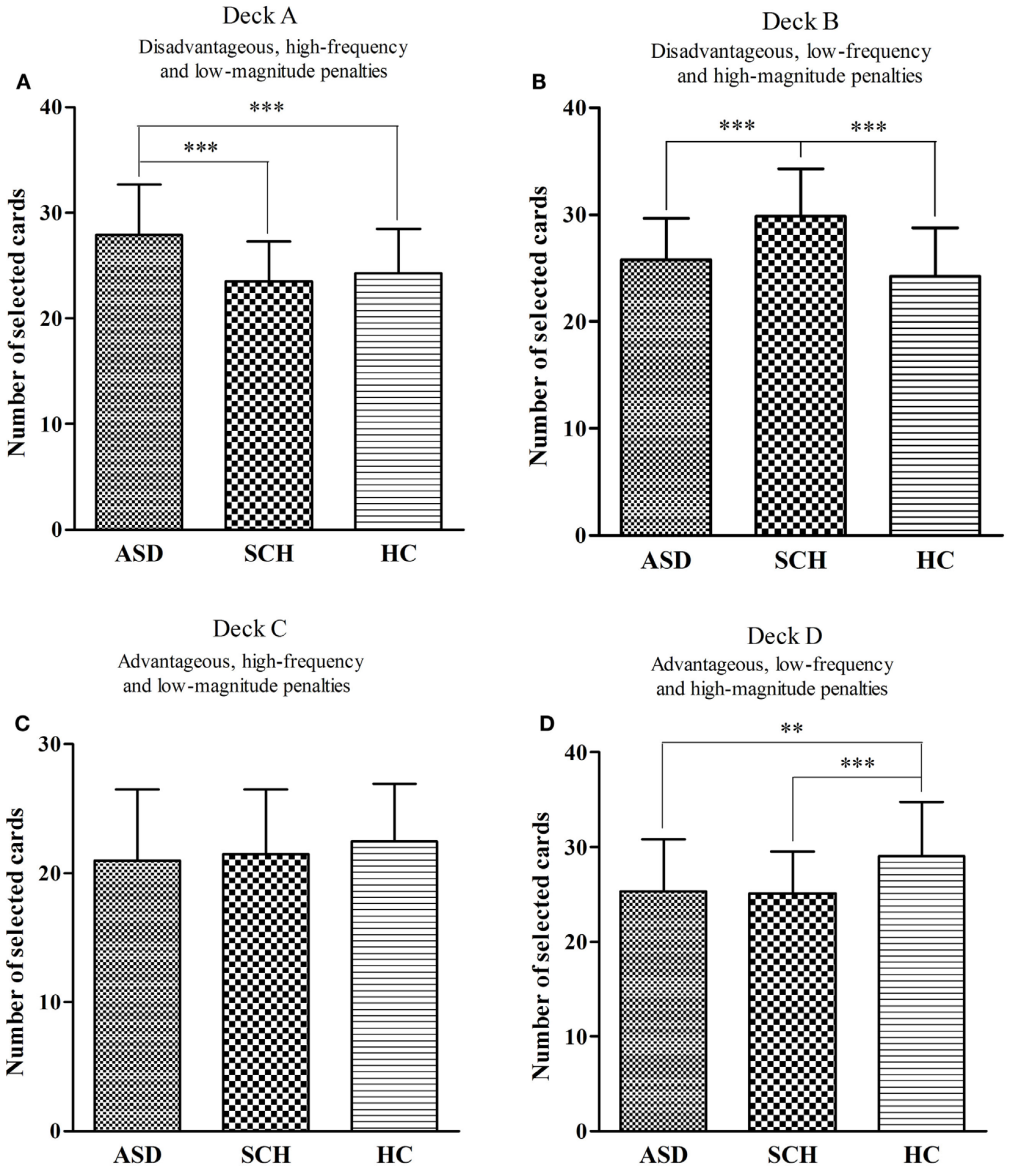
**Mean number of cards selected for participants with ASD, SCH, and HC from individual decks A (A), B (B), C (C), and D (D) during the IGT**. **p < 0.01 and ***p < 0.001. Mean ± SEM is shown.

There were significant differences in the overall score for deck A between the three groups [*F*(2,160) = 12.82, *p* < 0.001]. According to the *post hoc* Bonferroni-corrected comparisons, the ASD group selected significantly more cards from deck A than participants in the SCH and HC groups (all *p*s < 0.001, all *d*s ≥ 0.37), with no significant differences between the SCH and HC groups (*p* = 0.956, *d* = 0.10) (Figure 2A). There were significant differences in the overall score for deck B between the three groups [*F*(2,160) = 24.58, *p* < 0.001]. According to the *post hoc* Bonferroni-corrected comparisons, the SCH group selected significantly more cards from deck B than participants in the ASD and HC groups (all *p*s < 0.001, all *d*s ≥ 0.44), with no significant differences between the ASD and HC groups (*p* = 0.22, *d* = 0.18) (Figure 2B). There were no significant differences in the overall score for deck C between the three groups [*F*(2,160) = 1.41, *p* = 0.247] (Figure 2C). There were significant differences in the overall score for deck D between the three groups [*F*(2,160) = 10.73, *p* < 0.001]. According to the *post hoc* Bonferroni-corrected comparisons, the HC group selected significantly more cards from deck D than participants in the ASD and SCH groups (all *p*s ≤ 0.002, all *d*s ≥ 0.32), with no significant differences between the ASD and SCH groups (*p* = 1.000, *d* = 0.02) (Figure 2D).

**Figure 3:**
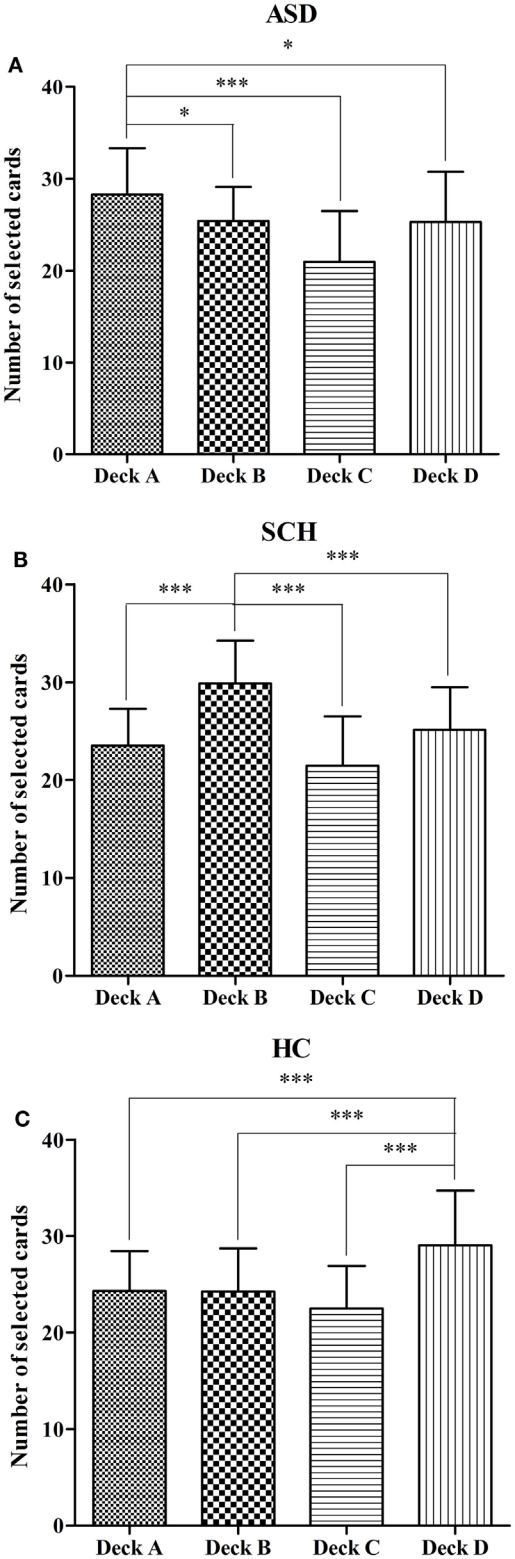
**Mean number of deck selections for individual decks A, B, C, and D in the participants with ASD (A), SCH (B), and HC (C) during the IGT**. *p < 0.05 and ***p < 0.001. Mean ± SEM is shown.

We also compared the number of selections for individual decks A, B, C, and D in each group. In the ASD group, the number of cards selected differed between the four decks [*F*(3,144) = 13.55, *p* < 0.001]. According to the *post hoc* Bonferroni-corrected comparisons, the ASD group selected significantly more cards from deck A than from decks B, C, and D (all *p*s < 0.01, all *d*s ≥ 0.27) (Figure 3A). In the SCH group, the number of cards selected differed between the four decks [*F*(3,180) = 29.96, *p* < 0.001]. According to the *post hoc* Bonferroni-corrected comparisons, the SCH group selected significantly more cards from deck B than from decks A, C, and D (all *p*s < 0.001, all *d*s ≥ 0.47) (Figure 3B). In the HC group, the number of cards selected differed between the four decks [*F*(3,316) = 28.36, *p* < 0.001]. According to the *post hoc* Bonferroni-corrected comparisons, the HC group selected significantly more cards from deck D than from decks A, B, and C (all *p*s < 0.001, all *d*s ≥ 0.43) (Figure 3C).

To examine how the individual deck level preference changes over the 100 trials, a repeated-measures ANOVA with deck as a between-subject factor and block as a within-subject factor was performed separately for each group. For the ASD group, there was a significant main effect for deck [*F*(3,144) = 12.80, *p* < 0.001], no significant main effect for block [*F*(4,576) = 0.00, *p* = 1.000], and an interaction effect [*F*(4,576) = 1.94, *p* = 0.028]. For the SCH group, there was a significant main effect for deck [*F*(3,180) = 29.96, *p* < 0.001], no significant main effect for block [*F*(4,720) = 0.00, *p* = 1.000], and an interaction effect [*F*(4,720) = 2.40, *p* = 0.005]. For the HC group, there was a significant main effect for deck [*F*(3,316) = 28.36, *p* < 0.001], no significant main effect for block [*F*(4,1264) = 0.003, *p* = 1.000], and an interaction effect [*F*(4,1264) = 9.23, *p* < 0.001].

**Figure 4:**
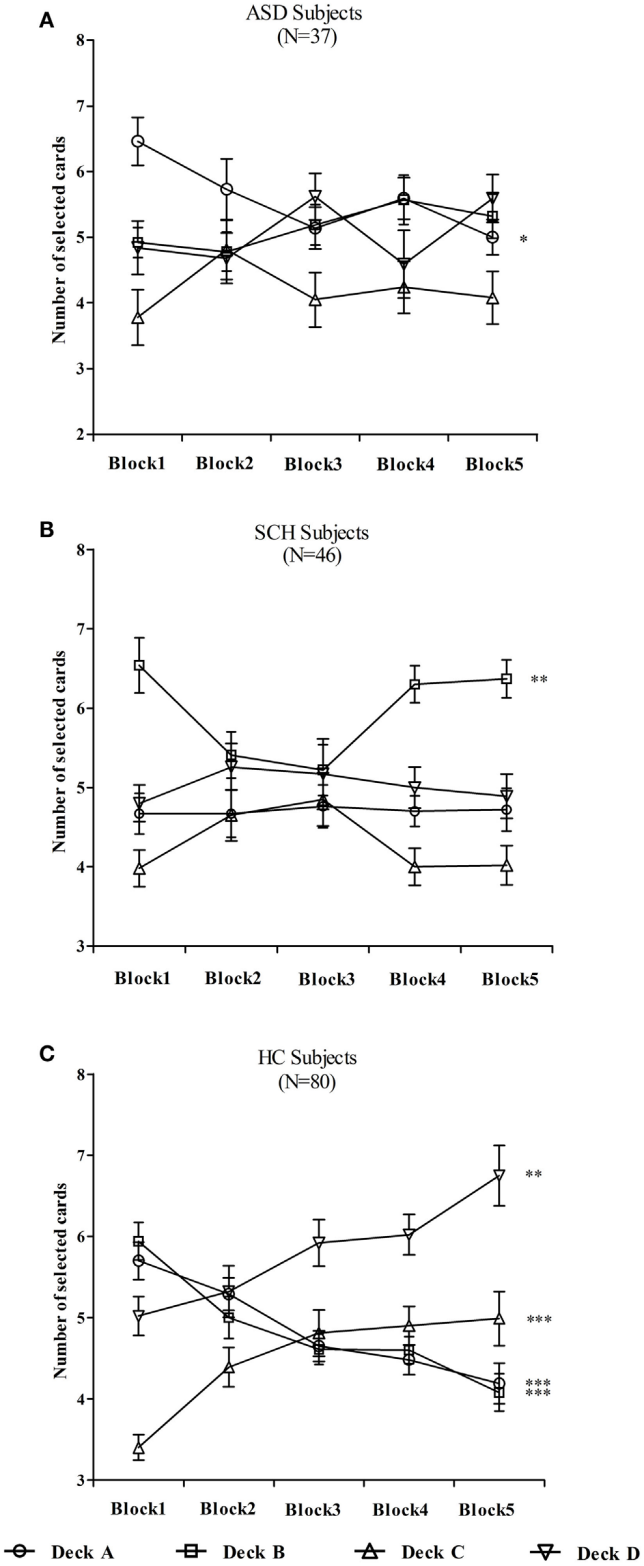
**Mean number of cards selected from individual decks A, B, C, and D for participants with ASD (A), SCH (B), and HC (C) during each block**. *p < 0.05, **p < 0.01, and ***p < 0.001. Mean ± SEM is shown.

In order to better understand the interactions, additional ANOVAs were computed to investigate which decks differed across the blocks. In the ASD group, the number of cards selected in deck A changed significantly over the five blocks [*F*(4,180) = 2.71, *p* = 0.032]. According to the *post hoc* Bonferroni-corrected comparisons, the number of deck A in block 1 was significantly more than that of block 5 (*p* = 0.037, *d* = 0.75). However, the number of cards selected did not change significantly in decks B, C, and D (all *p*s > 0.05) (Figure 4A). In the SCH group, the number of cards selected in deck B changed significantly over the five blocks [*F*(4,225) = 4.39, *p* = 0.002]. According to the *post hoc* Bonferroni-corrected comparisons, the number of deck B in block 1 was significantly more than that of block 3 (*p* = 0.014, *d* = 0.58). However, the number of cards selected did not change significantly in decks A, C, and D (all *p*s > 0.05) (Figure 4B). In the HC group, the number of cards selected changed significantly over the five blocks in decks A, B, C, and D (all *p*s < 0.01) (Figure 4C). According to the *post hoc* Bonferroni-corrected comparisons, the number of deck A in block 1 was significantly more than that of blocks 3, 4, and 5 (all *p*s ≤ 0.005, all *d*s ≥ 0.56), and the number of deck A in block 2 was significantly more than that of block 5 (*p* = 0.003, *d* = 0.54). The number of deck B in block 1 was significantly more than that of blocks 2, 3, 4, and 5 (all *p*s ≤ 0.024, all *d*s ≥ 0.43), and the number of deck B in block 2 was significantly more than that of block 5 (*p* = 0.027, *d* = 0.42). The number of deck C in block 1 was significantly less than that of blocks 3, 4, and 5 (all *p*s ≤ 0.001, all *d*s ≥ 0.68), and the number of deck D in blocks 1 and 2 was significantly less than that of block 5 (all *p*s ≤ 0.007, all *d*s ≥ 0.46).

### Decision-making on the GDT

#### Net Score of the GDT

**Figure 5:**
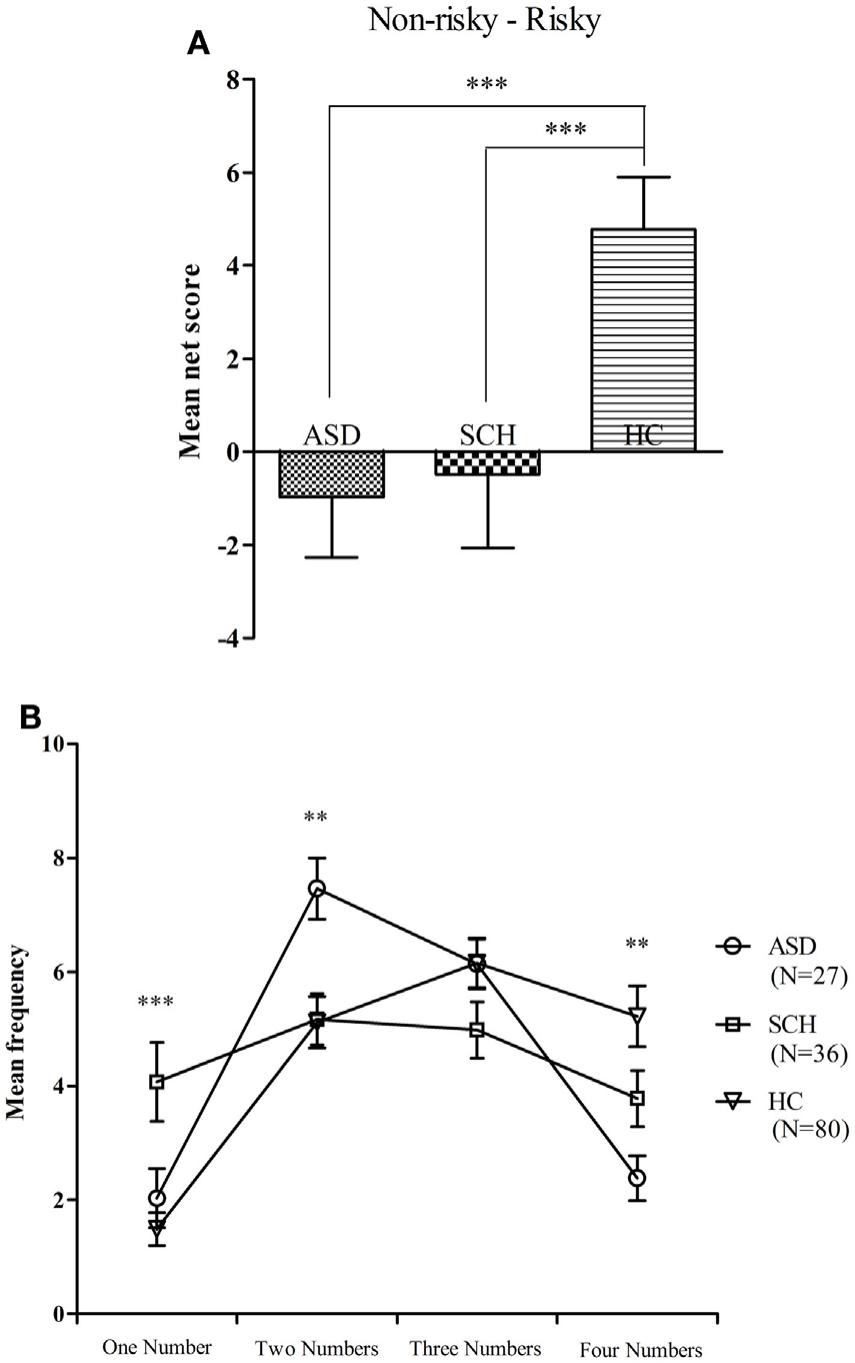
**Mean net score in the GDT (A) and mean number of choices in each single alternative (B) for subjects with ASD, SCH, and HC**. **p < 0.01 and ***p < 0.001. Mean ± SEM is shown.

There were significant differences between the GDT net score of the three groups [*F*(2,160) = 6.29, *p* = 0.002]. According to the *post hoc* Bonferroni-corrected comparisons, the HC group scored higher than the ASD and SCH groups (all *p*s ≤ 0.014, all *d*s ≥ 0.24), and there were no significant differences between the ASD and SCH groups (*p* = 1.000, *d* = 0.03) (Figure 5A). An ANOVA with repeated measures with alternative category as the within-subject factor and group as the between-subject factor was conducted. There was a main effect for choice [*F*(3,480) = 22.32, *p* < 0.001], and a significant interaction effect between alternative category and group [*F*(2,160) = 5.82, *p* < 0.001], but no main effect for group [*F*(2,160) = 0.00, *p* = 1.000].

The three groups significantly differed in the single choice [*F*(2,160) = 8.49, *p* < 0.001]. According to the *post hoc* Bonferroni-corrected comparisons, the SCH group chose more in the single choice than the ASD and HC groups (all *ps* ≤ 0.023, all *d*s ≥ 0.25), and there was no significant difference between the ASD and HC groups (*p* = 1.000, *d* = 0.09). The three groups significantly differed in the double choice [*F*(2,160) = 5.95, *p* = 0.003]. According to the *post hoc* Bonferroni-corrected comparisons, the ASD group chose more in the double choice than the SCH and HC groups (all *p*s ≤ 0.014, all *d*s ≥ 0.31), and there was no significant difference between the SCH and HC groups (*p* = 1.000, *d* = 0.01). The three groups significantly differed in the quadruple choice [*F*(2,160) = 6.82, *p* = 0.001]. According to the *post hoc* Bonferroni-corrected comparisons, the HC group chose more in the quadruple choice than the ASD (*p* = 0.001, *d* = 0.35), and there were no significant differences between the HC and SCH groups as well as no significant differences between the ASD and SCH groups (all *ps* ≥ 0.154, all *d*s ≤ 0.17). There were no significant differences between the three groups in the triple choice [*F*(2,160) = 1.87, *p* = 0.158] (Figure 5B). In a word, the HC group performed significantly better on the GDT than the ASD and SCH groups.

### Analyses about the use of feedback

We examined the use of negative feedback (losses) after the decision of a high-risk option to choose a low-risk option in the next trial; only those participants who chose a high-risk option and received negative feedback at least once during the GDT were included. Thus, the data of 150 subjects (ASD: *n* = 33; SCH: n = 41; HC: *n* = 76) were analyzed. The three groups differed on the use of negative feedback (%) [ASD: 36.16 ± 27.71; SCH: 52.02 ± 29.32; HC: 54.54 ± 36.26; *F*(2,147) = 3.75, *p* = 0.026]. According to the *post hoc* Bonferroni-corrected comparisons, the use of negative feedback in the ASD group was lower than in the SCH and HC groups (all *p*s < 0.05, all *d*s ≥ 0.27), and there were no significant differences between the SCH and HC groups (*p* = 1.000, *d* = 0.04).

We also examined the use of positive feedback (gains) after the decision of a low-risk option to choose a low-risk option again; only those participants who chose a low-risk option and received positive feedback at least once during the GDT were included. Thus, the analysis was based on the data of 155 participants (ASD: *n* = 35; SCH: *n* = 44; HC: *n* = 76). There were significant differences between the three groups with regard to the use of positive feedback (%) [ASD: 38.06 ± 25.85; SCH: 56.06 ± 27.15; HC: 53.00 ± 29.66; *F*(2,152) = 4.58, *p* = 0.012]. According to the *post hoc* Bonferroni-corrected comparisons, the use of positive feedback in the ASD group was lower than in the SCH and HC groups (all *p*s < 0.05, all *d*s ≥ 0.30), and there were no significant differences between the SCH and HC groups (*p* = 1.000, *d* = 0.05).

### Correlational analyses

Correlational analyses between the GDT performance and the neuropsychological measurements were examined. The use of negative feedback was significantly associated with the net score on the GDT in all three groups (ASD: *r* = 0.47, *p* = 0.006; SCH: *r* = 0.57, *p* < 0.001; HC: *r* = 0.25, *p* = 0.029). The use of positive feedback was also significantly associated with net score on the GDT in all three groups (ASD: *r* = 0.51, *p* = 0.002; SCH: *r* = 0.34, *p* = 0.023; HC: *r* = 0.37, *p* = 0.001). This means that the use of negative feedback and the use of positive feedback are associated with superior GDT performance.

The relationship between the net score on the GDT and performance on the WCST was examined. Perseverative errors were significantly associated with the net score on the GDT in all three groups (ASD: *r* = −0.39, *p* = 0.019; SCH: *r* = −0.33, *p* = 0.027; HC: *r* = −0.38, *p* < 0.001). There was a significant positive association between the categories completed and the net score on the GDT in all three groups (ASD: *r* = 0.36, *p* = 0.030; SCH: *r* = 0.46, *p* = 0.001; HC: *r* = 0.43, *p* < 0.001). This means that inferior performance on the WCST (high number of perseverative errors or low number of categories completed) is associated with inferior performance on the GDT.

The relationship between the net score on the GDT and performance on the TMT was examined. The TMT B was significantly associated with the net score on the GDT in all three groups (ASD: *r* = −0.48, *p* = 0.003; SCH: *r* = −0.31, *p* = 0.035; HC: *r* = −0.30, *p* = 0.006). There was a significant negative association between the difference score (TMT B − TMT A) and the net score on the GDT in all three groups (ASD: *r* = −0.52, *p* = 0.001; SCH: *r* = −0.43, *p* = 0.003; HC: *r* = −0.32, *p* = 0.004). This means that superior performance on the TMT [shorter time needed for TMT B or lower difference score (TMT B − TMT A)] is associated with superior performance on the GDT.

In general, performance on the GDT appears to be associated with executive functions and feedback processing in all three groups.

## Discussion

Two primary findings emerged from the present study. First, this study indicated that participants with ASD and SCH had impairments in both decision-making under ambiguity, as measured with the IGT, and decision-making under risk, as measured with the GDT. Second, the participants with ASD displayed a preference for deck A on the IGT, indicating that they showed more sensitivity to the magnitude of loss than to the frequency of loss, whereas the SCH patients displayed a preference for deck B, indicating that they showed more sensitivity to the frequency of loss than to the magnitude of loss. On the GDT, the impaired performance might be due to the deficits in executive functions in patients with SCH, whereas the impaired performance might be due to the deficits in feedback processing in participants with ASD.

In the present study, the subjects with ASD chose more disadvantageous decks than advantageous decks in the IGT. These findings were consistent with the study by Mussey et al. (2015), who found that the participants with ASD made worse decisions and showed slower learning with respect to which decks were advantageous compared with the HC. However, few studies until now have examined decision-making with a focus on reward/loss magnitudes or reward/loss frequencies (Damiano et al., 2012). The present study also found that participants with ASD had a preference for deck A, which had low-magnitude and high-frequency losses. These findings suggested that during the IGT, participants with ASD might be sensitive to the magnitude of loss but blind to the frequency of loss. It could be said that the occasional large loss associated with deck B was sufficient for the ASD group to attenuate their level of preference for this deck as compared to the patients with SCH who chose from deck B more often (South et al., 2014), that is, the ASD group showed more sensitivity to the magnitude than to the frequency of loss.

Similarly, consistent with the majority of the studies in the literature (Lee et al., 2007; Yip et al., 2009; Raffard et al., 2011; Fond et al., 2013; Brown et al., 2015), the performance on the IGT in the SCH patients was impaired compared to the performance of the healthy participants. Furthermore, our study found that patients with SCH displayed a stronger preference for selecting from deck B. These findings were consistent with previous studies that found that patients with SCH selected from the disadvantageous deck B significantly more frequently than they selected from deck A (Ritter et al., 2004; Shurman et al., 2005; Lee et al., 2007; Brown et al., 2015). A study using the expectancy-valence model also indicated that patients with SCH paid significantly greater attention to the smaller but more frequent losses in deck A and showed greater insensitivity to the intermittent large losses that occurred with deck B (Cella et al., 2012). That is, SCH patients showed more sensitivity to the frequency than to the magnitude of loss (Kim et al., 2009). In patients with SCH, the observation of a preference for the deck with rare large losses (deck B) appears to reflect a tendency to utilize outcome–frequency information at the expense of outcome–magnitude information. In other words, performance impairments on the IGT in SCH patients are more likely the result of a deficit in integrating frequencies and magnitudes of gains and losses (Brown et al., 2015).

The role of individual deck level preferences in assessing IGT performance was overlooked by most previous studies (Buelow and Suhr, 2013). Although decks A and B lead to long-term negative outcomes, deck A includes high-frequency and low-magnitude losses and deck B includes low-frequency and high-magnitude losses. Greater deck A or greater deck B selections depend on whether the subjects focus on the frequency or the magnitude of the loss (Bechara, 2008). Our findings provide some interesting implications for impairments in the IGT and emphasize the importance of examining selections from individual decks separately, as there were differences in the numbers of deck A and deck B selections between the participants with ASD and SCH in the current study. Therefore, combining decks A and B into a disadvantageous deck may mask individual deck level preference (Buelow and Suhr, 2013).

Through analysis of the GDT score, it was found that participants with ASD displayed impaired performance on the GDT. To the best of our knowledge, this study was the first to use the GDT to examine decision-making under risk in participants with ASD. One important and new finding in the current study is that GDT performance among participants with ASD was significantly related to impaired performance on negative and positive feedback. In other words, performance on the GDT was significantly associated with feedback processing in participants with ASD, as was previously observed in healthy individuals (Brand, 2008; Yao et al., 2014) and other clinical populations (Labudda et al., 2009, 2010). Feedback processing on the GDT refers to using a loss (negative feedback) after a high-risk decision to choose a safe option and using a gain (positive feedback) after a low-risk decision to choose a safe option again. However, in our study, the feedback analysis indicated that the abilities of participants with ASD to use negative feedback after a high-risk decision and positive feedback after a low-risk decision were strongly reduced. Therefore, we suggested that the impaired performance on the GDT might be due to the deficits in feedback processing in participants with ASD.

The SCH patients also differed from the HC in their higher number of choices of high-risk options. Our findings were consistent with those of Fond et al. (2013), who found that individuals with SCH have globally impaired decision-making capacities. Moreover, our study showed that the GDT performance in SCH patients was significantly correlated with executive functions as measured by the WCST and TMT, as was previously found in other patient groups (Euteneuer et al., 2009; Labudda et al., 2009). More significantly, our results showed that patients with SCH demonstrated inferior executive functions as compared to participants with ASD and HC. Therefore, we inferred that the impaired performance on the GDT might be due to the deficits in executive functions in patients with SCH.

Brand et al. (2006) proposed that there may be two interacting routes that can be used to guide decision-making under risk as measured with the GDT: a cognitive route, in which information about consequences and probabilities is integrated and utilized before a decision is made, and an emotional route, in which feedback in terms of gains and losses is processed. In line with the proposed cognitive route, the impaired performance on the GDT might be due to the deficits in executive functions in patients with SCH, and in agreement with the proposed emotional route, the impaired performance on the GDT might be due to the deficits in feedback processing in participants with ASD.

With respect to the DST, our study found that the ASD group demonstrated normal DST performance compared with the HC group. This finding was in accordance with previous studies on the DST (Nakahachi et al., 2006; Cui et al., 2010). The normal digit span would indicate normal ability of verbal working memory in the participants with ASD. However, some studies showed that ASD were impaired in visual spatial working memory (Goldberg et al., 2005; Luna et al., 2007). These results might reveal imbalance of working memory development in individuals with ASD. In our study, performance of the SCH group on the DST was comparative with that of the HC group. This result is inconsistent with some studies that found reduced DST performance in SCH patients (Sánchez-Morla et al., 2009; Brébion et al., 2014). Patients in most of the previous studies were receiving antipsychotic medications at the time of testing. However, patients with SCH in the present study had never been treated with any antipsychotic medications. Moreover, most of the patients with SCH in these studies were older adults (Brébion et al., 2014), while SCH patients in our study were adolescents and young adults. These two points may help explain the discrepancy.

Our findings of overlapping features of impairments in decision-making suggest that ASD and SCH might share a neural basis, particularly in systems related to mechanisms of social functions. However, exploring the salient differences in cognitive mechanisms of deficits in decision-making between these two disorders is more reasonable and significant. Although comparative designs have several defects, they offer an effective and simple method for discovering shared and divergent mechanisms underlying pathways to social or cognitive dysfunction (Sasson et al., 2011) and providing new insights and perspectives into disorder-specific prevention and treatment methods (Hommer and Swedo, 2015).

## Conclusion

In summary, our study revealed that individuals with ASD and SCH displayed impairments both in decision-making under ambiguity and in decision-making under risk. However, these impairments might involve different cognitive mechanisms in the two disorders. Our findings may possibly support the notion that different mechanisms underlie similar social dysfunctions in ASD and SCH. Additional studies utilizing both neuroimaging and genetic techniques are warranted to explore the common and differing neurobiological processes involved in decision-making in participants with ASD and SCH.

## Author Contributions

LZ was primarily responsible for the design and conduct of the study, the analysis of the data, and writing the first draft of the manuscript. JT, YD, YJ, YW, ZL, and JC played a role in subject recruitment and contributed to data collection. RT and LZ conducted statistical analyses and supervised work with subjects. KW was the Research Coordinator of the project, designed the study, and wrote the protocol. All authors have revised and approved the final manuscript.

## Conflict of Interest Statement

The authors declare that the research was conducted in the absence of any commercial or financial relationships that could be construed as a potential conflict of interest.

## Abbreviations

ANOVA, analysis of variance; AQ, Autism-Spectrum Quotient; ASD, autism spectrum disorders; DSM, *Diagnostic and Statistical Manual*; DST, Digit Span Test; GDT, Game of Dice Task; HC, healthy controls; IGT, Iowa Gambling Task; SCH, schizophrenia; TMT, Trail Making Test; WCST, Wisconsin Card Sorting Test.
